# A Combination of Anatolian Propolis and Curcumin Protects Fibroblasts Against Beclomethasone (Nazal Steroid)-Induced Oxidative Stress by Modulating IL-25, MMP-2, VEGF, and FGF-2 Expressions

**DOI:** 10.3390/ph18030326

**Published:** 2025-02-26

**Authors:** Tarik Yagci, Sidika Genc, Riza Dundar, Halil Ibrahim Altiner, Ali Taghizadehghalehjoughi

**Affiliations:** 1 Department of Otorhinolaryngology, Faculty of Medicine, Şeyh Edebali University, 11230 Bilecik, Turkey; tarik.yagci@bilecik.edu.tr (T.Y.); dundarkbb2@gmail.com (R.D.); dribrahimaltiner@gmail.com (H.I.A.); 2 Department of Medical Pharmacology, Faculty of Medicine, Şeyh Edebali University, 11230 Bilecik, Turkey; sidika.genc@bilecik.edu.tr

**Keywords:** Anatolian propolis, curcumin, IL-25, MMP-2, VEGF, BCM

## Abstract

**Background:** Nasal steroids are commonly prescribed in ear, nose, and throat clinics. It is observed that the use of nasal steroids is increasing due to the prevalence of allergic rhinitis. Because beclomethasone (BCM) toxicity is low, it is highly preferred in allergic rhinitis. The rate of toxicity increases with the increase in the duration and dose of BCM use. However, the protective mechanism of Anatolian propolis (AP) and curcumin (Cur) against BCM toxicity has not been fully explained. **Aim:** The study evaluates the potential BCM-induced toxicity effect on VEGF, MMP-2, IL-25, and IL-10 parameters after Cur and AP treatment. **Materials and Methods:** Cell viability, oxidative stress, and gene expression were used for toxicity evaluation. **Results:** AP 2.5 mg/mL and Cur 16 µg/mL show high viability and antioxidant capacity. BCM increased the levels of IL-25, IL-10, and MMP-2, and a decrease was detected in the expression levels of FGF-2 and VEGF. **Conclusions:** AP and Cur show effective healing, and AP has been shown to improve inflammation more effectively than Cur. However, the combination of AP and Cur significantly improved the induced toxicity effects.

## 1. Introduction

Nasal steroids (NS) are very commonly prescribed in ear, nose, and throat clinics. They are preferred before and after surgery in chronic rhinosinusitis (CRS), allergic rhinitis (AR), and turbinate hypertrophies. It is observed that the use of NS is increasing due to the prevalence of AR in the world being 10–20% [1] and the prevalence of chronic rhinosinusitis being 11% [2]. Commonly, NS toxic effects occur with the unconscious use or overdose of nasal steroids. Different studies have revealed that the resulting damage causes a delay in wound healing, epistaxis, nasal mucosal atrophy, septal perforation, cell stress rupture, deterioration in vascular structures, and apoptosis [3,4]. Therefore, in order not to limit the use of NS but to reduce toxicity, in our study, the effects on the toxicity model of using propolis (from the Anatolia region) and Cur were investigated. The main purpose of our study is to use AP and Cur to eliminate the negative effects and, thus, minimize these side effects in our work with systems that allow long-term use.

Especially in nasal epithelial wound healing; fibroblast growth factor 2 (FGF-2) is involved in cell proliferation, vascular endothelial growth factor (VEGF) is involved in the formation of new vascular lines, and interleukin-25 (IL-25) and matrix metalloproteinase (MMP) are important in reducing the inflammatory process [5,6,7]. VEGF and FGF-2 are powerful angiogenetic growth factors in wound healing. With the release of angiogenic substances, important processes such as the activation, adhesion, and migration of endothelial cells begin via MMP-2 [8,9]. Additionally, it has been shown in different studies that IL-25 contributes to the wound-healing process by supporting fibroblast activation [10,11,12].

Propolis is a resinous honeybee product obtained from beehives as raw material [13]. Propolis shows changes in chemical composition when comparing samples taken from different places and even from the same place, depending on its botanical origin. Despite chemical differences, it is well known that samples of different geographical origins and chemical compositions generally show similar biological activity. The main components of propolis are wax, resin, and volatile substances. However, the biological activity of propolis is attributed to plant-derived substances. Propolis is a protective agent against microorganisms in the hive.

In studies conducted with propolis in various nasal wounds or wounds caused by different reasons, it was found that it significantly increased and healed VEGF and FGF-2 levels [4,14,15,16]. AP contains a higher amount of phenol compounds than other types of propolis [17], and it is known that this substance is effective in wound healing by preventing oxidative damage and nitrosative stress in the human body [18,19] and increasing the release of various growth hormones [20,21,22].

Curcumin is a plant extract from the ginger family. Cur has an anti-inflammatory and antioxidant effect by suppressing the expression of tumor necrosis factor-alpha (TNF-α) and reactive oxygen species (ROS) [23]. These effects lead to an increase in remodeling and the proliferative process, increasing wound healing [24,25,26]. In addition, it has been shown that Cur significantly increases wound healing in nasal mucosal damage in rats, and this is due to increased angiogenesis with the release of MMP-2 and VEGF [27,28,29].

Our study will demonstrate, for the first time, using AP and Cur, pure and combined, to improve the toxic effects of NS on VEGF, FGF-2, MMP-2, and IL-25 pathways. Honeybees produce AP. Propolis collected by honeybees by visiting different plants shows a wide range of biological activities (antioxidant effects). Curcumin is obtained from the turmeric plant and has been reported to have high antioxidant effects. The combined use of AP and Cur can have synergistic anti-inflammatory and antioxidant effects on the toxicity caused by BMC. To investigate this situation, two different dose-combination groups were investigated in the study. These four different pathways will be examined after 24 h using the in vitro nasal fibroblast wound model created in this way.

## 2. Results

### 2.1. GC/MS

**Figure 1:**
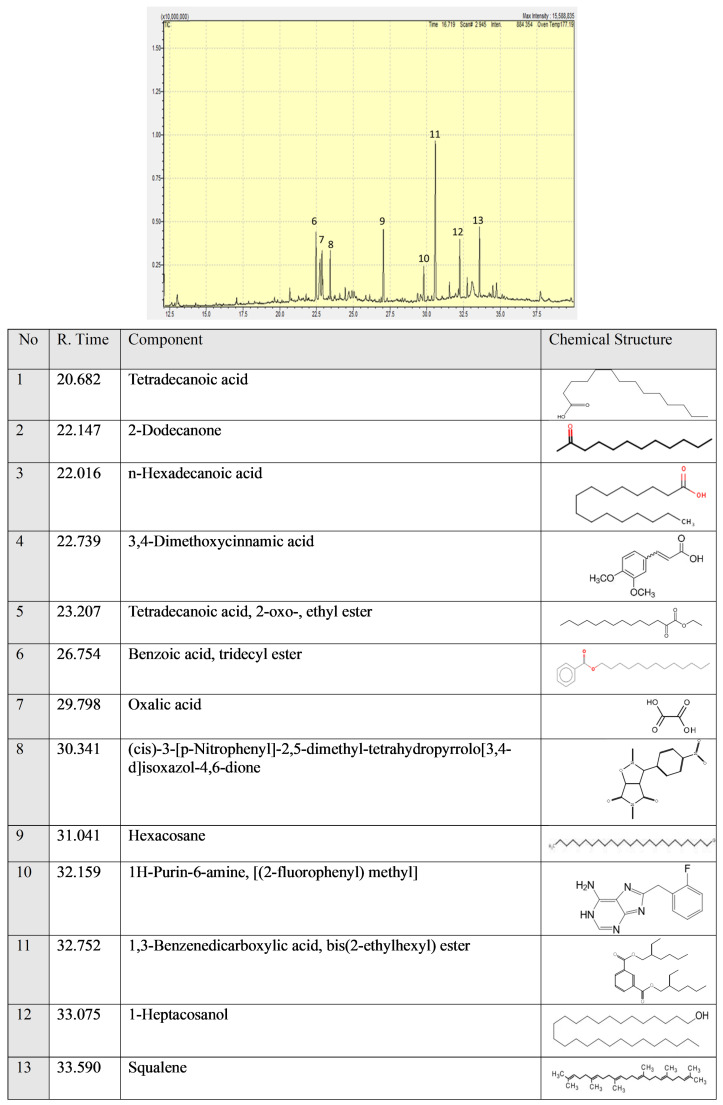
The AP GC/MS spike and ingredients list.

The GC/MS results are shown in Figure 1. Thirteen ingredients were detected. Squalene is a very important terpenoid among volatile isolates. 3,4-Dimethoxycinnamic acid is a cinnamic acid derivative that has an anti-inflammatory effect. 1-heptacosanol has been reported in the essential oil of Hibiscus sabdariffa flowers (found in the Anatolian region).

### 2.2. MTT Assay Result

**Figure 2:**
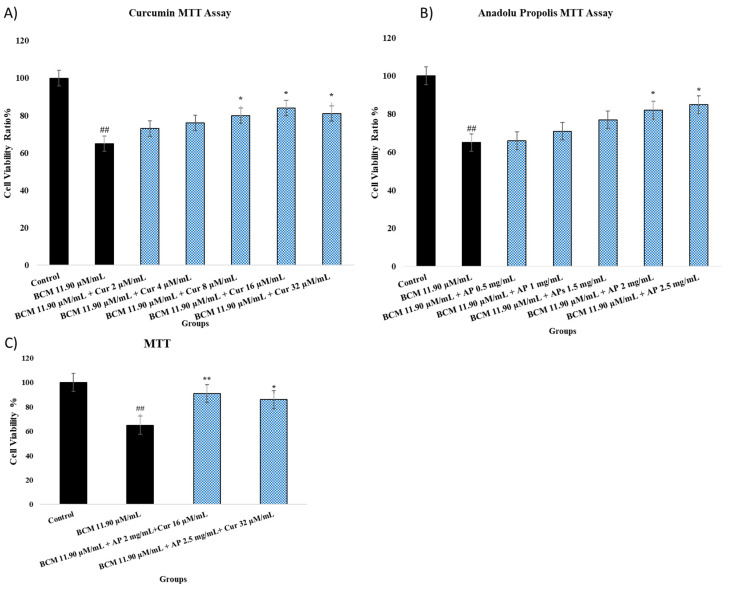
(**A**,**B**) show the cell viability ratio of nasal fibroblast 24 h after treatment. (**A**) shows Cur’s protective effects against BCM-induced toxicity. The experimental groups contain the control group, BCM 11.90 µM/mL + Cur 2 µM/mL, BCM 11.90 µM/mL + Cur 4 µM/mL, BCM 11.90 µM/mL + Cur 8 µM/mL, BCM 11.90 µM/mL + Cur 16 µM/mL, and BCM 11.90 µM/mL + Cur 32 µM/mL. (**B**) shows AP protective effects against BCM-induced toxicity. The experimental groups contain the control group, BCM 11.90 µM/mL + AP 0.5 mg/mL, BCM 11.90 µM/mL + AP 1 mg/mL, BCM 11.90 µM/mL + AP 1.5 mg/mL, BCM 11.90 µM/mL + AP 2 mg/mL, and BCM 11.90 µM/mL + AP 2.5 mg/mL. (**C**) shows the combination group protective effects against BCM-induced toxicity. The experimental groups contain the control group, BCM 11.90 µM/mL, BCM 11.90 µM/mL AP 2 mg/mL + Cur 16 µM/mL, and BCM 11.90 µM/mL AP 2.5 mg/mL + Cur 32 µM/mL. Statistical significance: ^##^ *p* < 0.001 compared to the control group. * *p* < 0.05; ** *p* < 0.001 compared to the BCM group.

The cell viability results are shown in Figure 2A–C. Cell viability results were obtained in the 24th hour after toxicity induction and received treatments. The groups formed by the use of the BCM IC_50_ dose as a toxicity model, (A) BCM, Cur, (B) BCM and AP, and (C) combination groups, were evaluated. The control group was rated as 100 and the viability rate of the other groups was evaluated compared to the control group. First, the BCM group was compared with the control group, and the treatment groups were statistically compared with the BCM group. BCM shows significant differences in compression compared to the control at a rate of *p* < 0.001.

As shown in Figure 2A, with the increase in treatment group doses, statistical differences were seen in comparison to the control group. It was observed that the use of Cur at doses of 8, 16, and 32 µg/mL increased the viability statistically at a rate of *p* < 0.05 compared to the BCM group. Figure 2B illustrates that AP at doses of 2 and 2.5 µg/mL resulted in a slight increase in the viability ratio (*p* < 0.05 compared to BCM). Cell viability in combination groups AP 2 µg/mL + Cur 16 µM/mL and AP 2.5 µg/mL + Cur 32 µM/mL was detected at 91% (*p* < 0.001) and 86% (*p* < 0.05), respectively.

### 2.3. TAC and TOS Assay

**Figure 3:**
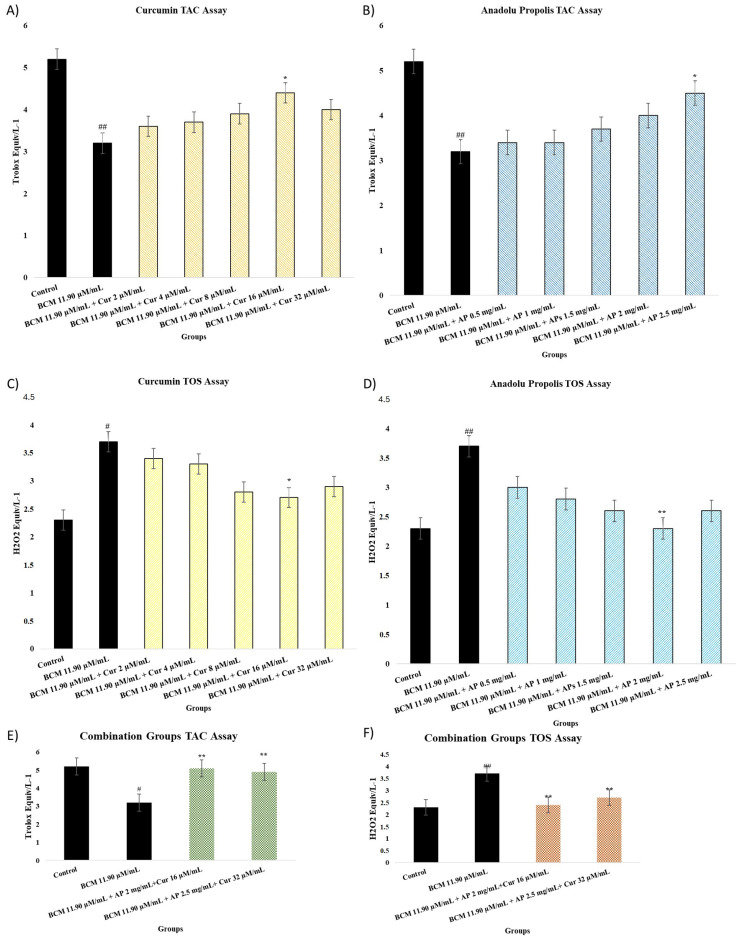
(**A**–**F**) show the total antioxidant capacity and total oxidant capacity of nasal fibroblast 24 h after treatment by Cur and AP. (**A**,**C**,**E**) show Cur TAC and TOS assay results. The experimental groups contain the control group, BCM 11.90 µM/mL + Cur 2 µM/mL, BCM 11.90 µM/mL + Cur 4 µM/mL, BCM 11.90 µM/mL + Cur 8 µM/mL, BCM 11.90 µM/mL + Cur 16 µM/mL, BCM 11.90 µM/mL + Cur 32 µM/mL, BCM 11.90 µM/mL AP 2 mg/mL + Cur 16 µM/mL, and BCM 11.90 µM/mL AP 2.5 mg/mL + Cur 32 µM/mL. (**B**,**D**,**F**) show AP TAC and TOS assay results. The experimental groups contain the control group, BCM 11.90 µM/mL + AP 0.5 mg/mL, BCM 11.90 µM/mL + AP 1 mg/mL, BCM 11.90 µM/mL + AP 1.5 mg/mL, BCM 11.90 µM/mL + AP 2 mg/mL, BCM 11.90 µM/mL + AP 2.5 mg/mL, BCM 11.90 µM/mL AP 2 mg/mL+ Cur 16 µM/mL, and BCM 11.90 µM/mL AP 2.5 mg/mL + Cur 32 µM/mL. Statistical significance: ^##^ *p* < 0.001, ^#^ *p* < 0.05 compared to the control group. * *p* < 0.05; ** *p* < 0.001 compared to the BCM group.

The evaluation results regarding TAC and TOS results are shown in Figure 3A–D. TAC (A, B) and TOS (C, D) results were obtained at the end of 24 h exposure to BCM (11.90 µM/mL), Cur (2, 4, 8, 16, and 32 µM/mL), AP (0.5, 1, 1.5, 2, and 2.5 mg/mL), and combination treatment (AP 2 mg/mL + Cur 16 µg/mL and AP 2.5 mg/mL + Cur 32 µg/mL). In the study, the BCM group was evaluated according to the control group, and the treatment groups were evaluated according to the BCM group. It is observed that the use of BCM (11.90 µM/mL) decreased TAC values (*p* < 0.001) while a significant increase was seen in TOS levels (*p* < 0.001). Figure 3A shows that Cur 16 µg/mL resulted in the largest increase in the TAC value compared to other Cur doses (*p* < 0.05). An increase in the TAC value was seen only in the AP 2.5 mg/mL group (*p* < 0.05, Figure 2B).

From Figure 3C, it can be seen that Cur decreased the TOS value (*p* < 0.05) at a dose of 16 µg/mL in comparison to the BCM group. When the dose of Cur was increased to 32 µg/mL, no statistical significance was observed. Figure 3D shows that significant decreases were seen only when the AP dose was 2 mg/mL (*p* < 0.001).

The AP 2 mg/mL + Cur 16 µg/mL and AP 2.5 mg/mL + Cur 32 µg/mL combination groups results show that the TAC value was increased compared to the BCM group (*p* < 0.001). Whereas, TOS levels show a significant decrease compared to the BCM group (Figure 3E,F).

### 2.4. LDH and GR Assay

**Figure 4:**
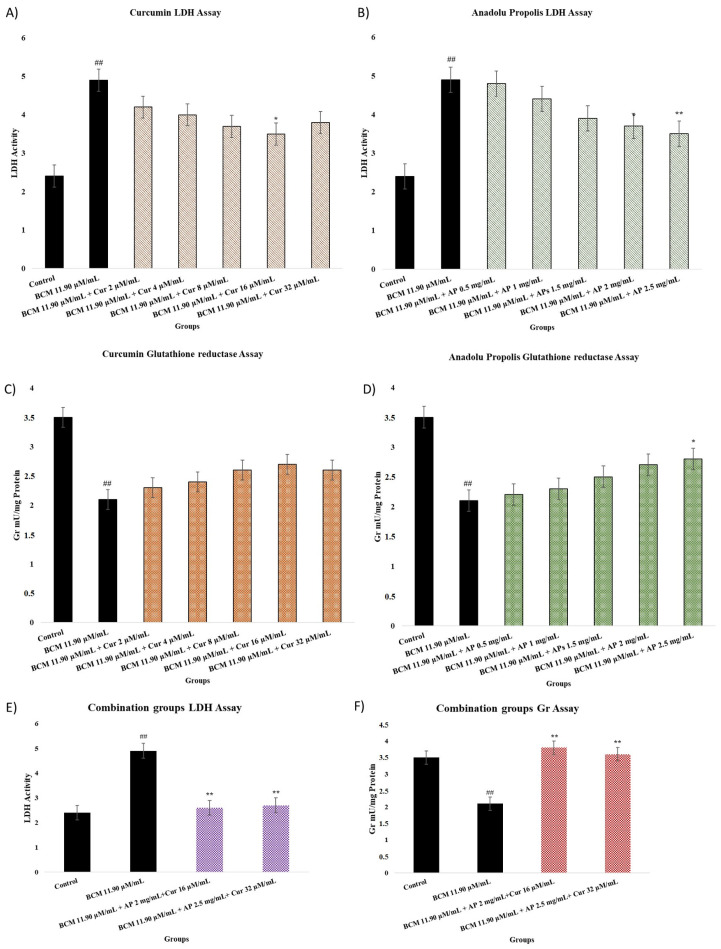
(**A**–**F**) show LDH and Gr levels of nasal fibroblast after treatment by Cur and AP for 24 h. (**A**,**C**,**E**) show Cur LDH and Gr assay results. The experimental groups contain the control group, BCM 11.90 µM/mL + Cur 2 µM/mL, BCM 11.90 µM/mL + Cur 4 µM/mL, BCM 11.90 µM/mL + Cur 8 µM/mL, BCM 11.90 µM/mL + Cur 16 µM/mL and BCM 11.90 µM/mL + Cur 32, BCM 11.90 µM/mL AP 2 mg/mL + Cur 16 µM/mL, and BCM 11.90 µM/mL AP 2.5 mg/mL + Cur 32 µM/mL µM/mL. (**B**,**D**) show AP LDH and Gr assay results. The experimental groups contain the control group, BCM 11.90 µM/mL + AP 0.5 mg/mL, BCM 11.90 µM/mL + AP 1 mg/mL, BCM 11.90 µM/mL + AP 1.5 mg/mL, BCM 11.90 µM/mL + AP 2 mg/mL, and BCM 11.90 µM/mL + AP 2.5 mg/mL. Statistical significance: ^##^ *p* < 0.001 compared to the control group. * *p* < 0.05; ** *p* < 0.001 compared to the BCM group.

The evaluation results regarding LDH and GR results are shown in Figure 4A–F. LDH and GR tests were done after 24 h exposure to BCM (11.90 µM/mL), Cur (2, 4, 8, 16, and 32 µg/mL), AP (0.5, 1, 1.5, 2, and 2.5 mg/mL), AP 2 mg/mL + Cur 16 µM/mL, and AP 2.5 mg/mL + Cur 32 µM/mL are shown. The BCM group statically was compared with a control group and the treatment groups were compared to the BCM group. It is observed that BCM increased LDH values (*p* < 0.001) (A, B, and E) and, at the same time, decreased GR levels, respectively (*p* < 0.001) (C, D, and F). As shown in Figure 3A, Cur antioxidant properties caused a decrease in LDH levels but it was not significant (*p* > 0.05). It can be seen the AP 2 and 2.5 mg/mL doses decreased LDH levels (Figure 3B) by *p* < 0.05 and *p* < 0.001, respectively. For AP 2 mg/mL + Cur 16 µM/mL 2.6 and AP 2.5 mg/mL + Cur 32 µM/mL 2.7, activity of LDH was detected (*p* < 0.001).

From Figure 4C, there is a visible increase in GR values with the use of BCM and Cur in increasing doses. This increase was not statistically significant in any of the doses of Cur used. Considering Figure 4D, GR values increase with the use of BCM and AP in increasing doses. However, this increase does not create a statistically significant difference in any of the AP doses. AP 2 mg/mL + Cur 16 µM/mL 3.8 Gr mU/mg protein and AP 2.5 mg/mL + Cur 32 µM/mL 3.6 Gr mU/mg protein were measured (*p* < 0.001).

### 2.5. IL-25 and IL-10 Assay

**Figure 5:**
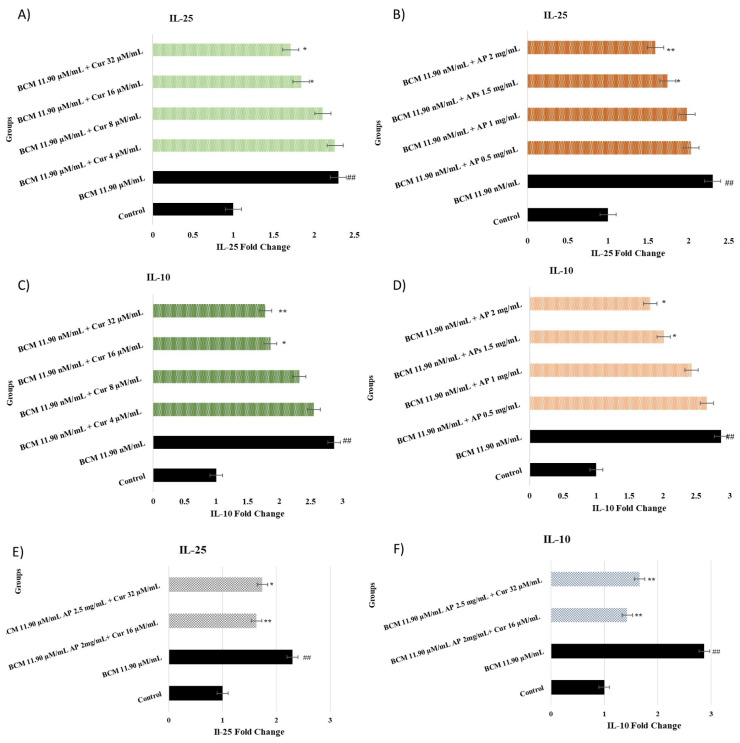
(**A**–**F**) show Il-25 and IL-10 expression fold change of nasal fibroblast after treatment by Cur and AP. (**A**,**C**) show Cur IL-25 and IL-10 assay results. The experimental groups contain the control group, BCM 11.90 µM/mL + Cur 4 µM/mL, BCM 11.90 µM/mL + Cur 8 µM/mL, BCM 11.90 µM/mL + Cur 16 µM/mL, and BCM 11.90 µM/mL + Cur 32 µM/mL. (**B**,**D**) show AP IL-25 and IL-10 assay results. The experimental groups contain the control group, BCM 11.90 µM/mL + AP 1 mg/mL, BCM 11.90 µM/mL + AP 1.5 mg/mL, BCM 11.90 µM/mL + AP 2 mg/mL, and BCM 11.90 µM/mL + AP 2.5 mg/mL. Statistical significance: ^##^ *p* < 0.001 compared to the control group. * *p* < 0.05; ** *p* < 0.001 compared to the BCM.

The evaluation results regarding IL-25 and IL-10 results are shown in Figure 5A–D. Control groups were rated as 1 and the other groups were proportioned. The BCM group was compared to the control group. Then, the treatment groups were compared to the BCM group. It is observed that the IL-25 and IL-10 values in the cells increase statistically significantly (*p* < 0.01) with the use of BCM (A, B, C, D). From Figure 4A, a decrease in IL-25 value is observed with the use of Cur 32 µg/mL (*p* < 0.05), while AP more effectively decreased IL-25 expression (2, 2.5 mg/mL, *p* < 0.05 and *p* < 0.001, respectively).

Figure 5C illustrates that Cur more effectively decreased IL-10 values in 16 and 32 µg/mL doses (*p* < 0.05 and *p* < 0.001, respectively). Decreased IL-10 expression level only in the 2.5 mg/mL AP group was significant (*p* < 0.05).

### 2.6. MMP-2, HBD, FGF-2, and VEGF Assay

**Figure 6:**
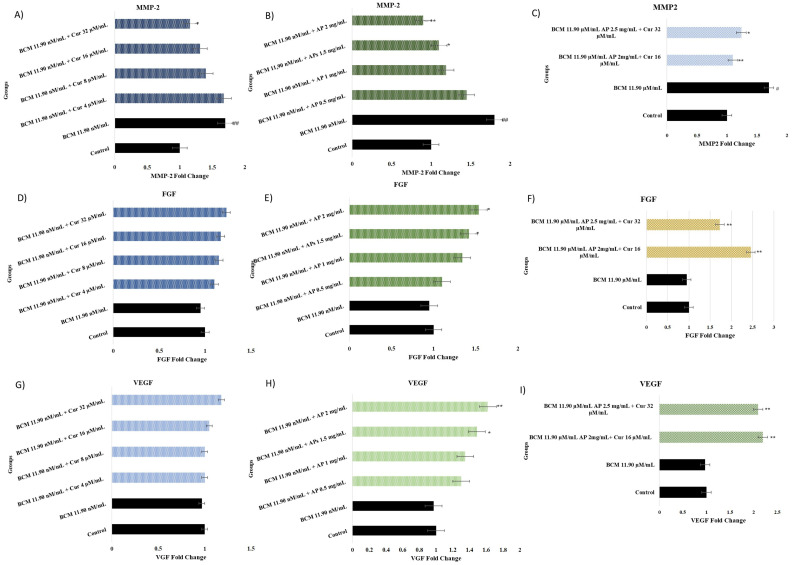
(**A**–**I**) show MMP-2 (**A**,**B**), FGF-2 (**C**,**D**), and VEGF (**E**,**F**) expression fold change of nasal fibroblast after treatment by Cur and AP. The experimental groups contain the control group, BCM 11.90 µM/mL, BCM 11.90 µM/mL + Cur 4 µM/mL, BCM 11.90 µM/mL + Cur 8 µM/mL, BCM 11.90 µM/mL + Cur 16 µM/mL and BCM 11.90 µM/mL + Cur 32 µM/mL, BCM 11.90 µM/mL + AP 1 mg/mL, BCM 11.90 µM/mL + AP 1.5 mg/mL, BCM 11.90 µM/mL + AP 2 mg/mL, and BCM 11.90 µM/mL + AP 2.5 mg/mL. Statistical significance: ^##^ *p* < 0.001, ^#^ *p* < 0.05, * *p* < 0.05; ** *p* < 0.001 compared to the BCM group.

The evaluation results regarding MMP-2, HBD, FGF-2, and VEGF results are shown in Figure 6A–F. HBD gene expression for both treatments was NA. In this study, evaluations of MMP-2 (A, B), FGF-2 (C, D), and the VEGF (E, F) tests were performed for groups: control group, BCM (11.90 µM/mL), Cur (4, 8, 16, and 32 µg/mL), and AP (1.5, 2, and 2.5 mg/mL). The BCM group statically was compared with a control group and the treatment groups were compared to the BCM group. It is observed that with the use of BCM, MMP-2, FGF-2, and VEGF values in the cells decreased but this is not a significant difference (*p* > 0.05).

Figure 6A,B show that a visible increase in the MMP-2 value was seen in the BCM group (*p* < 0.001). MMP-2 levels decreased significantly after treatment by Cur 32 µg/mL (*p* < 0.05), and the 2–2.5 mg/mL AP dose (*p* < 0.05, *p* < 0.001, respectively). Figure 5C,D show that although there is a visible increase in FGF-2 values with the Cur treatment, the increase is not statistically significant (*p* > 0.05). AP 2 and 2.5 mg/mL increased FGF-2 expression levels up to 49 and 62%, respectively (*p* < 0.05, *p* < 0.001).

In Figure 6E,F, VEGF values were not statistically different in Cur groups. AP increased the VEGF expression level in 2 mg/mL (*p* < 0.05) and 2.5 mg/mL (*p* < 0.001) doses.

### 2.7. IL-25, IL-10, MMP-2, FGF-2, and VEGF Elisa Assay

**Figure 7:**
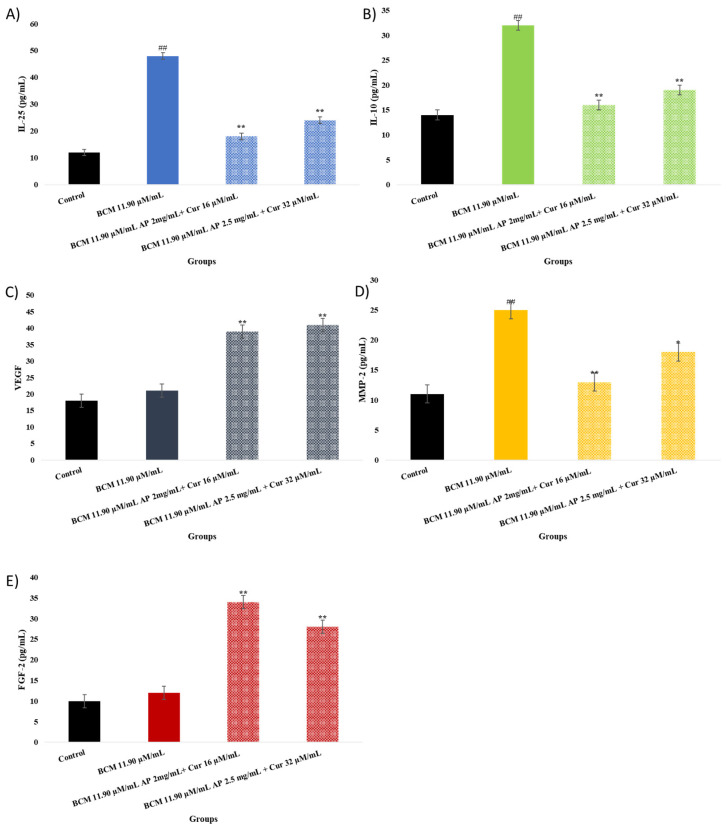
(**A**–**E**): (**A**) IL-25 protein level; (**B**) IL-10 protein level; (**C**) VEGF protein level; (**D**) MMP-2 protein level; (**E**) FGF-2 protein level in fibroblast cell culture. The experimental groups contain the control group, BCM 11.90 µM/mL, BCM 11.90 µM/mL AP 2 mg/mL + Cur 16 µM/mL, and BCM 11.90 µM/mL AP 2.5 mg/mL + Cur 32 µM/mL. ^##^ *p* < 0.001, ** *p* < 0.001 and * *p* < 0.05 compared to the control group.

The evaluation results regarding IL-25, IL-10, MMP-2, FGF-2, and VEGF Elisa Assay results are shown in Figure 7A–E. Protein levels of IL-25, IL-10, and MMP-2 were observed to be higher than in the control group (*p* < 0.001), whereas FGF-2 and VEGF protein levels did not show any significant difference compared to the control group. The combination group’s protein levels show differences in comparison to the BCM control group. However, the AP 2 mg/mL + Cur 16 µM/mL combination group in the MMP-2 test did not show a significant difference compared to the BCM group (*p* < 0.05).

## 3. Discussion

Corticosteroids form the basis of the treatment of asthma and allergies. Corticosteroids, as regulators of the immune system, suppress the apoptosis of eosinophils and lymphocytes and the production of inflammatory eicosanoids and cytokines. However, unconscious use of corticosteroids has been shown in different studies to cause tissue damage. BCM is regularly and widely used in ear, nose, and throat diseases. Although BCM causes less toxicity than other corticosteroids, studies have shown that its toxicity causes a decrease in cell viability, oxidative stress damage, a decrease in vascularization, a decrease in cell proliferation, and an increase in inflammation when used in high doses [3,4,30,31].

A BCM IC_50_ dose of 11.90 µM/mL was used. In an in vitro study, it was shown that the BCM IC50 dose inhibited the viability of fibroblasts [32]. In our study, we observed that the viability (MTT) of nasal fibroblast cells decreased by 35% after the use of BCM (11.90 µM/mL) and this rate was statistically significant (*p* < 0.01) (Figure 1). Corticosteroids change protein synthesis in target tissues. They interact with corticosteroid receptors on the cell membrane to form a steroid-receptor complex. This complex creates a difference in second messenger stimulation and DNA expression [33]. The study showed that Cur increased cell viability in fibroblasts [34]. An increase in the cell viability ratio of intestinal epithelial cells was shown after Cur (5 µg/mL) administration [35]. In addition, Cur (0.1–20 μM doses) treatment for 48 h caused an increase in the cell viability ratio (*p* < 0.01) [31]. In our study, different doses of Cur (8, 16, and 32 µM/mL) were applied to nasal fibroblast cells after treatment with BCM. It was determined that cell viability increased significantly compared to the BCM group. Similarly, there is evidence that propolis species increase cell viability in fibroblasts [36,37]. It was observed that AP (5 μg/mL) increased cell viability in nasal epithelial cells, although it was not statistically significant [4]. In our study, it was observed that cell viability increased significantly (*p* < 0.05) with the use of AP (2 and 2.5 mg/mL). In addition, a synergy effect was seen in the combination of Ap and Cur mainly due to antimicrobial activities, antioxidant, and anti-inflammatory effects [38].

Squalene has anti-inflammation and wound-healing effects. Squalene is responsible for AP protective effects. We observed a statistically significant (*p* < 0.05) increase in MTT values using AP (2 and 2.5 mg/mL). Cinnamic acid derivatives 3,4-Dimethoxycinnamic acid have antiproliferative activity against tumors [39]. 1-heptacosanol has been reported to have antimicrobial and antioxidant activity [40] and antibacterial activity [41].

The maintenance of cell viability is directly related to the balance between antioxidant capacity and oxidative damage in the cell [42]. The oxidant and antioxidant levels were determined using TAC and TOS analyses. Glucocorticoids have been shown to increase oxidative damage in cells [43,44]. However, there are also publications showing that Cur and Propolis cause significant changes in the values of many oxidative stress markers by reducing oxidative damage in the cell [45,46]. In an in vitro study by Waly MI et al., Cur (1 mM) brought the TAC values of kidney cells exposed to cisplatin up to the level of the control group [47]. In our study, although Cur (16 mM) did not increase the TAC level as much as the control group, there was a statistically significant (*p* < 0.05) difference.

Many previous studies have shown that propolis increases the TAC value and decreases the TOS value. However, there are no published studies in the literature on AP’s oxidative stress damage markers in the cell. In our study, we showed that AP (2.5 mg/mL) reduced the TAC level at a statistically significant rate (*p* < 0.05), and when AP (2 mg/mL) was used, it decreased the TOS level at a statistically significant rate (*p* < 0.01). A study tried to evaluate the antifibrotic effect of curcumin, N-acetyl cysteine, and propolis extract against bisphenol A-induced hepatotoxicity in rats. They showed that the antioxidant and anti-inflammatory effects of these three ingredients protect the liver from induced toxicity [48]. In addition, we obtain better results in the in vitro study due to Anatolian propolis.

Li X et al., [49] in their in vitro study, showed that the increased LDH level in the cell exposed to oxygen-glucose deprivation/reoxygenation was reduced to a statistically significant extent (*p* < 0.01) by using Cur 5 and 10 μM. It has also been shown to increase glutathione levels significantly (*p* < 0.01). In another study, Cur’s ability to reduce the toxic effects of herbicides was evaluated. It has been revealed that there is a positive correlation between Cur and GR levels [36]. In our study, combination groups more effectively decreased LDH levels and increased GR levels. In addition, all doses of Cur act not effectively but the same as combination groups. The decrease in the LDH level was statistically significant when Cur was used at a dose of 16 μM.

The study showed that propolis can reduce oxidative stress and inflammation in rheumatoid arthritis disease in women. The results of a clinical trial showed that a reduction in oxidative stress and cytokines improved symptoms [50]. A study by Joanna Kocot et al. showed that propolis contains many plant-derived flavonoid and phenolic components [51]. Bees visit many different flowers because the components formulate propolis antioxidative stress and antioxidant capacity more strongly than many natural products. According to our results, the propolis effects on LDH, TAC, TOS, and Gr results are more effective than those of Cur.

Inflammation occurring in fibroblast cells leads to changes in the bronchial epithelium and lung microenvironment [52]. Various inflammatory and pro-inflammatory markers such as transforming growth factor-beta (TGF-β), interleukin 6 (IL-6), interleukin 10 (IL-10), and interleukin 25 (IL-25) activate signal transduction and transcription activator [44,45]. Subsequent STAT3 phosphorylation plays an important role in the regulation of angiogenic mediators such as VEGF, FGF-2, and HIF1, leading to angiogenesis [53]. In light of the findings from our study, it was determined that BCM caused significant differences in VEGF and FGF-2 gene levels. We think that this effect is especially effective through the STAT-3 pathway and drags the cells into inflammation and the apoptotic path. In addition, reactive oxygen species and the PI3K/AKT signaling pathway are also responsible for the expression of FGF-2. BCM-induced toxicity activated the redox system of the cells, and the apoptotic process was activated with the increase in oxidative damage. To eliminate BCM-induced damage, we investigated the effects of Cur and AP separately in the treatment groups. In the current study, Cur decreased IL-25 and IL-10 levels at a high dose of 32 µM/mL and a low dose did not show any difference in comparison to the BCM group. The protective effects of AP at 2 and 2.5 mg/mL were more significant than the cur groups.

MMP-2 shows an increase especially in inflammation as a regulatory modulator. Barbara Fingleton et al. investigated MMP-2’s role in chronic and acute inflammation. The MMP-2 level shows an increase in response to inflammation to start regeneration [54]. This mechanism is a protective mechanism. The MMP-2 result in the BCM group shows an increase but treatment with AP more effectively decreased MMP-2. We think propolis’ different phenolic components have a role in low MMP-2 levels.

## 4. Materials and Methods

### 4.1. Chemicals and Reagents

All the reagents were of analytical grade and used without further purification. AP (Bee and You company, Nazobec (Farmamag İlaç San ve Tic A.Ş., Turkey)), Dulbecco-modified eagle medium (DMEM), fetal bovine serum (FBS), Antibiotic, and dimethyl sulfoxide (DMSO) were purchased from Sigma Aldrich (St. Louis, MO, USA). Primers and regents for gene expression and cDNA analyses by real-time PCR were obtained from Roche (Darmstadt, Germany).

### 4.2. GC/MS Spectrometry

Shimadzu/QP2010-ultra (Tokyo, Japan) was used for the analysis of the AP sample. Experimental conditions of the GCMS system were as follows. A REX 5MS column (30 m × 0.25 mm, 0.25 μm film thickness) was used. The column flow rate of the mobile phase (He) was set at 1 mL/min. In the GC part, the temperature was kept for 3 min at 40 °C and then increased to 250 °C at 10 °C/min intervals followed by 5 min at 150 °C. Finally, the temperature was increased to 300 °C at 18 °C/min for 42 min. The mass range (*m*/*z*) was determined as 30–500. The experiment was conducted in the Central Research Laboratory Application and Research Center (BARUM, Bilecik, Turkey)

### 4.3. Nasal Fibroblast Cell Culture

The fibroblast cell line (PCS-201-012, ATCC) was obtained from Bilecik University, Department of Medical Pharmacology. Cells were centrifuged at 1200 rpm for 5 min. They were suspended in fresh Dulbecco-modified eagle medium-F12 (DMEM-F12), fetal bovine serum (FBS) 10%, and antibiotics 1% (penicillin, streptomycin, and amphotericin B). Then, 48-well plates (5% CO_2_; 37°) were used for seeding.

### 4.4. MTT Assay

Briefly, cells were resuspended in fresh DMEM medium, 10% FBS, and 1% antibiotic (penicillin, streptomycin, and amphotericin B). Cells were then seeded in 96-well plates (Corning, NY, USA) as previously described and stored in an incubator (5% CO_2_; 37 °C). After reaching 85% confluence on a 0.5 McFarland scale, the experiment was conducted with 14 groups. For the BCM toxicity model, BCM 11.90 µM/mL was exposed for 30 min; then, the treatment was added to the plate and the experiment was conducted for 24 h. The sample size was 8 (*n* = 8). The control group received only medium and the BCM control group received BCM 11.90 µM/mL [32]. BCM 11.90 µM/mL + AP 0.5 mg/mL, BCM 11.90 µM/mL + AP 1 mg/mL, BCM 11.90 µM/mL + AP 1.5 mg/mL, BCM 11.90 µM/mL + AP 2 mg/mL, BCM 11.90 µM/mL + AP 2.5 mg/mL, BCM 11.90 µM/mL + Cur 2 µM/mL, BCM 11.90 µM/mL + Cur 4 µM/mL, BCM 11.90 µM/mL + Cur 8 µM/mL, BCM 11.90 µM/mL + Cur 16 µM/mL, and BCM 11.90 µM/mL + Cur 32 µM/mL was administered for 24 h. Combination groups BCM 11.90 µM/mL + AP 1.5 mg/mL, BCM 11.90 µM/mL + AP 2 mg/mL, and BCM 11.90 µM/mL + AP 2.5 mg/mL for evaluation of a potential synergy effect were added for 24 h. At the end of the experiment, 10 μL of MTT solution (Sigma Aldrich, St. Louis, MO, USA) was added to each well plate and the samples were incubated for 4 h; 100 μL of DMSO (Millipore Sigma) was added to all wells to dissolve the Formazan crystals. The optical density of the solutions was read at 570 nm using a Multiskan™ GO microplate spectrophotometer (Thermo Fisher, Porto Salvo, Portugal) [55].

### 4.5. TAC (Total Antioxidant Capacity) and TOS (Total Oxidant Status)

TAC and TOS evaluation was performed using a supernatant medium of each group at the end of the study. TAC level was measured by using the Rel Assay Total Antioxidant Capacity (Rel Assay Diagnostics, Gaziantep, Turkey) commercial kit. Briefly, the supernatant was used and the reagent was added according to the manufacturing protocol. The color change was evaluated by measuring at a wavelength of 660 nm for TAC and 530 nm for TOS. TAC results were expressed per µmol Trolox Equiv/mg protein. TOS results were expressed per µmol H_2_O_2_ Equiv/mg protein [56].

### 4.6. LDH (Lactate dehydrogenase) and Gr (Glutathione reductase) Assay

LDH and Gr evaluation was performed using lysed cells of each group at the end of the study. The cells were lysed by 0.5% triton-x and then centrifuged at 5.000× *g* for 5 min to obtain the supernatant. The supernatants were taken and used for analysis. Lactate dehydrogenase (LDH) and Gr (Glutathione reductase) levels were assessed using the ELISA kit (Elabscience, United States Cat no., Houston, TX, USA) according to the manual of the manufacturer. At 450 nm, the optical densities of each sample were measured.

### 4.7. Real-Time PCR Analysis

#### 4.7.1. RNA Isolation

mRNA isolation was obtained according to the procedure of Invitrogen™ Total RNA Isolation Kit (Catalog number: 4478545).

#### 4.7.2. Gene Expression

Gene expression levels were examined in the effective groups. MMP-2, IL-25, VEGF, and IL-10 genes, 0.25 μL of right and left primer, 0.15 μL of the probe, 3 μL of cDNA, 3 master mix, and 12.75 μL of distilled water were added to each strip (tube) at this stage. The final volume was adjusted to 20 μL. After 600 s at 95 °C, 10 s at 95 degrees, and 30 s at 60 degrees, 45 cycles were made [57].

β-actin

Forward: 5′-CCAACCGCGAGAAGATGA-3′

Reverse: 5′-CCAGAGGCGTACAGGGATAG-3′

MMP-2

Forward: 5′- CCGAGGACTATGACCGGGATAA-3

Reverse: 5′- CTTGTTGCCCAGGAAAGTGAAG-3′

IL-25

Forward: 5′-TGGCAATGATCGTGGGAACC-3′

Reverse: 5′-GAGAGATGGCCCTGCTGTTGA-3′

VEGF

Forward: 5′-ACCATGAACTTTCTGGTCTCTTG-3′

Reverse: 5′-TCGGGGTACTCCTGGAAGATG-3′

IL-10

Forward:5′-GGCGCTGTCATCGATTTCTT-3′

Reverse: 5′-TCTCTTGGAGCTTATTAAAGGCATTC-3′

FGF-2

Forward:5′-GTTGACCCTACCATGTTCCCTTG-3′

Reverse: 5′-GCCAGCAGCATCTATGGGAC-3′

HBD

Forward:5′-GACAGGTACGGCTGTCATCA-3′

Reverse: 5′-CAGCCTAAGGGTGGGAAAAT-3′

### 4.8. ELISA Test: Klotho, Galanin, mTOR, and STAT-3

The fibroblast cells were washed in iced PBS (pH 7.4) to thoroughly remove the supernatant residue. The cells were lysed and centrifuged at 12,000 RPM at 4 °C for 15 min. IL-25, IL-10, MMP-2, FGF-2, and VEGF BTLAB ELISAs (Cat No: E0054Hu, E0102Hu, E0315Ra, E1592Mo, and E0114Mo) were performed according to the kit protocol and each sample’s optical density was measured at 450 nm [58].

### 4.9. Statistical Analysis

Data were statistically defined as mean and standard deviation (mean ± SD) for % area. One-way ANOVA and Tukey tests (SPSS 22.0) were performed to compare positive immunoreactive cells and immunopositive stained areas with healthy controls. As a result of the test, a value of *p* < 0.05 was considered significant and the data were presented as mean ± SD.

## 5. Conclusions

In our study, we evaluated MTT, TAC, TOS, LDH, and Gr parameters to evaluate whether BCM reduces cellular viability and increases oxidative stress in nasal fibroblasts. We used Cur and AP as treatments to reverse these effects. As seen so far, we have shown that cell viability is increased, and oxidative damage is prevented with the treatments we provide. We used the genetic analysis parameters IL-25, IL-10, MMP-2, FGF-2, HBD, and VEGF. Considering the results obtained, while BCM increased IL-25, IL-10, and MMP-2 levels, a decrease was detected in the expression levels of FGF-2 and VEGF. Contrary to these data, although AP and Cur show effective healing, AP has effectively decreased inflammation. Future Directions: This study is limited to in vitro analyses. In future studies, combined treatments against BCM toxicity (AP 2 µg/mL + Cur 16 µM/mL) can be validated with in vivo experiments and their clinical applicability can be tested.

## Data Availability

The dataset presented in this study is available from the corresponding author upon reasonable request.
